# Supplementary material for: Transcriptome Analysis of Two Tetraploid Potato Varieties under Water-Stress Conditions
Source: Int J Mol Sci. 2022 Nov 11;23(22):13905. doi: 10.3390/ijms232213905 (PMC9694765; doi:10.3390/ijms232213905)
Supplement: Supplementary file 1 [file ijms-23-13905-s001.zip › ijms-2008026-supplementary.pdf]

Table S1. Sequences of primers used in RT-qPCR analysis.

GENE ID	Primer sequence
**LOC102594541**	Forward: ACTGTGTGAGCAGATTCGGG<br>Reverse: ATATCTAAGGCGCTGACGGC
**LOC102584727**	Forward: AATGGCTGGTGGGGGTGAT<br>Reverse: CTCTGTGAGCCAAGTGCCAA
**LOC102599586**	Forward: GCCGACTGTTCTCTTCCACC<br>Reverse: AGTTTGAATCCACTCCCTGCTG
**LOC102606222**	Forward: CGCAGCTAACAATCCGACCT<br>Reverse: TAGTGGGCGTCATTTCGTCG
**LOC102593738**	Forward: ATCCTCATCCACAAAGAACACCAA<br>Reverse: TGCTGCTCCTGCCAATCTGT
**LOC102580220**	Forward: AACAACAATAACTACATGCCGAGC<br>Reverse: TTAGATGCAAGTGACAGGCCCAA
**LOC107062634**	Forward: GCTCCTGTGGTGTTTTTCAGGTAA<br>Reverse: TCATATCCATCAGCAACTCGACC
**LOC102606295**	Forward: TGCTTTTGCTGGACTTTTAGGTATG<br>Reverse: GCACGCTTCCACTTAGACCA
**LOC102600114**	Forward: TTTGCGTAAGTCCCCCGTC<br>Reverse: CCTCTCTCGCTCAATATCCTTTTCA
**LOC102598238**	Forward: AGCAGTACCCTATTCCTCCTCG<br>Reverse: CACAAATCTTTCTTGCCTGCCC
**LOC102603161**	Forward: TACTGGTGCTACTGGATTTCTTGC<br>Reverse: GTCCTTTGCCACTGCCTCAT
**LOC102601774**	Forward: TTGGGTTCATCACTGGACTACAC<br>Reverse: CTTTTGCAGGATCTACGAAGGGT
**Tubulin**	Forward: GGGAATAACTGGGCGAAAGGT<br>Reverse: CCTCCACCAAGTGAGTGACAA
